# Ambiguous genitalia: clinical management of adult female with male assigned gender: a case report

**DOI:** 10.1186/s13256-021-02914-2

**Published:** 2021-07-12

**Authors:** Mahamudu Ayamba Ali, Raymond Saa-Eru Maalman, Yaw Otchere Donkor, James Edward Mensah

**Affiliations:** 1 Department of Basic Medical Sciences, School of Medicine, University of Health and Allied Sciences, Ho, Volta Region Ghana; 2 Consultant Urologist, Department of Surgery, School of Medicine, University of Health and Allied Sciences, Ho, Volta Region Ghana; 3 Consultant Urologist, Department of Surgery, Korle Bu Teaching Hospital, Accra, Ghana; 4 School of medicine and Dentistry, University of Ghana, College of Health Sciences, Accra, Ghana

**Keywords:** Disorders of sex development, Ambiguous genitalia, Clinical management, Surgical approach, Adult female

## Abstract

**Background:**

Disorders of sex development are anomalies in which the development of urogenital ridge is undifferentiated for the male and female child. Imaging plays a vital role in investigating the gross anatomy and associated anomalies. Ultrasonography, such as genitography and magnetic resonance, is the primary modality for demonstrating internal gonads and genitalia. Early multidisciplinary approach in the management of ambiguous genitalia including early surgical intervention is the predominant practice, with few current considerations on deferral of genital reconstruction until adolescent age.

**Case presentation:**

We report the rare case of a 24-year-old adult female from a majority ethnic group of the Volta region, Ghana who was diagnosed and raised as male, now requiring surgical restoration to the female gender. The surgical team decided to assign external genitalia to correspond with the already intact internal organs, thus constructing the vulva. Consent was given by the client and her family members for management and surgical intervention. The surgery was scheduled and duly performed with a successful outcome. Understanding and consent was sought from the patient for the purpose of using her images for teaching, scientific publication, and demonstrations.

**Conclusion:**

The advantages of deferring surgical reconstruction with psychological counseling after early assessment need to be considered to prevent inappropriate gender assignment.

## Background

The term ambiguous genitalia describes the most common clinical presentation of a variety of congenital conditions classified as disorders of sexual development (DSD) or differences in sex development [1, 2]. DSDs are associated with atypical development of the internal and external genital structures as a result of variations in genes, developmental programming, and hormones [3]. DSDs occur when chromosomal, gonadal, or anatomical sex is atypical [4, 5], and before 2006, DSD was termed as intersex [2]. Based on the karyotype, three main groups of DSDs are identified using the new nomenclature proposed by the Chicago Consensus (Table 1).

**Table 1:** Disorders of sex development: Chicago Classification of sex [4]

Sex chromosome DSD	46, XY DSD	46, XX DSD
45, XO (Turner syndrome and variants)	Disorders of gonadal (testicular) development Complete gonadal dysgenesis (Sawyer syndrome) Partial gonadal dysgenesis, gonadal regression, ovotesticular DSD	Disorders of gonadal (ovarian) development Ovotesticular DSD Testicular DSD (SRY+, dup SOX9) • Gonadal dysgenesis
47, XXY (Klinefelter syndrome and variants)	Disorders in androgen synthesis or action • Androgen biosynthesis defect (17-hydroxysteroid dehydrogenase deficiency, 5α-reductase deficiency)	Androgen excess Fetal (21-or 11-hydroxylase deficiency)
45, X/46, XY (mixed gonadal dysgenesis, ovotesticular DSD)	Defect in androgen action (complete androgen insensitivity syndrome)	Fetoplacental (aromatase deficiency, P450 oxidoreductase)
46, XX/46, XY (chimeric, ovotesticular DSD)	LH receptor defects (Leydig cell hypoplasia) Disorders of AMH and AMH receptor (persistent Müllerian duct syndrome)	Maternal (luteoma, exogenous)

DSD: Disorders of Sex development, LH: Luthenizing hormone, AMH: Antimulerian hormone, SRY: Sex region Y chromosome

Disorders of sex development are a major pediatric issue, accounting for approximately 1% of all live births [5]. However, the incidence varies between developed and developing countries [6]. In Ghana, there is a paucity of information, but in other developing countries such as Saudi Arabia incidence is reported as 1 in 2500 live births and in Egypt 1 in 3000 live births [2, 6]. In a developed country such as Germany, the incidence is as low as 2 per 10,000 live births. The high rate of DSD was hypothesized to result from higher consanguinity among populations of developing countries than among those from developed countries [2]. The underlying cause of ambiguous genitalia in a newborn/child needs extensive and urgent investigation to avoid missing a life-threatening problem such as congenital adrenal hyperplasia (CAH), which is a major presentation of DSD [2].

Clinical management of DSDs is carried out per the classification proposed by the Paediatric Endocrinology Society Lawson Wilkins and the European Society of Paediatric Endocrinology [7]. The management of patients with DSD and related conditions is focused on four aspects: (1) etiological diagnosis, (2) assignment of gender, (3) indication for and timing of genital surgery, and (4) disclosure of medical information to the patient [8]. Thorough clinical, hormonal, radiological, chromosomal, and molecular evaluations are therefore essential. However, a prompt evaluation of these assignments may be challenging for accurate diagnosis and appropriate therapy, especially in resource-deprived countries. Satisfactory management of these children does not merely demand early diagnosis and appropriate treatment, but gender assignment will produce a positive impact on the outcome. Failure of the appropriate approach causes great discomfort for family and healthcare providers [9].

Genetic tools such as microarray analyses and next-generation sequencing techniques have identified novel genetic variants among patients with DSD. Most importantly, patient management needs to be individualized especially for decisions related to the sex of rearing, surgical interventions, hormone treatment, and potential for fertility preservation [3]. In countries with an inadequate health system, there are challenges in the diagnosis as well as management of DSDs, as in the case reported herein.

## Case presentation

**Fig. 1:**
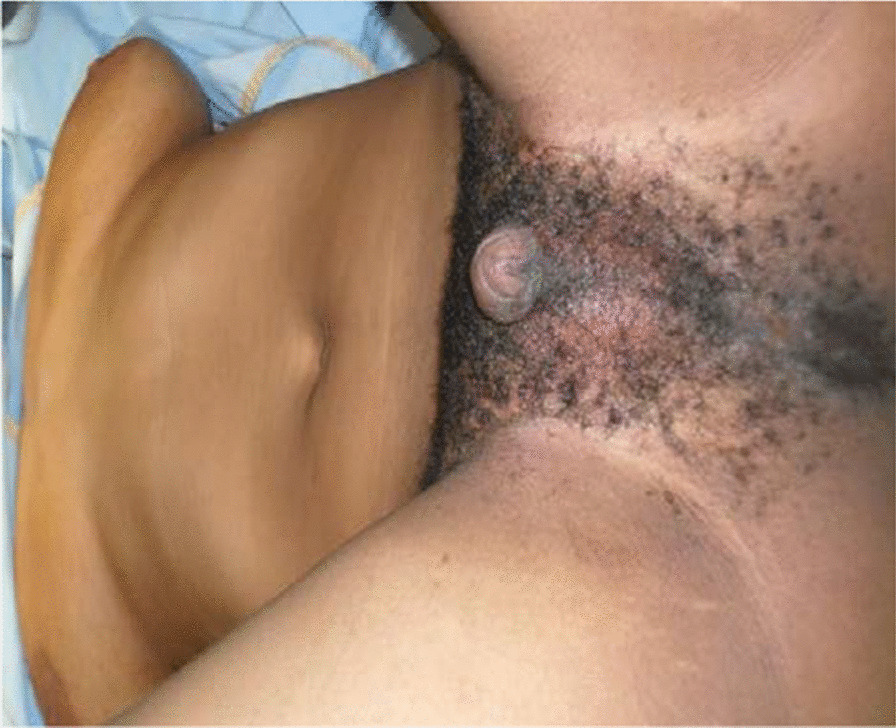
A mega clitoris without vaginal opening

**Fig. 2:**
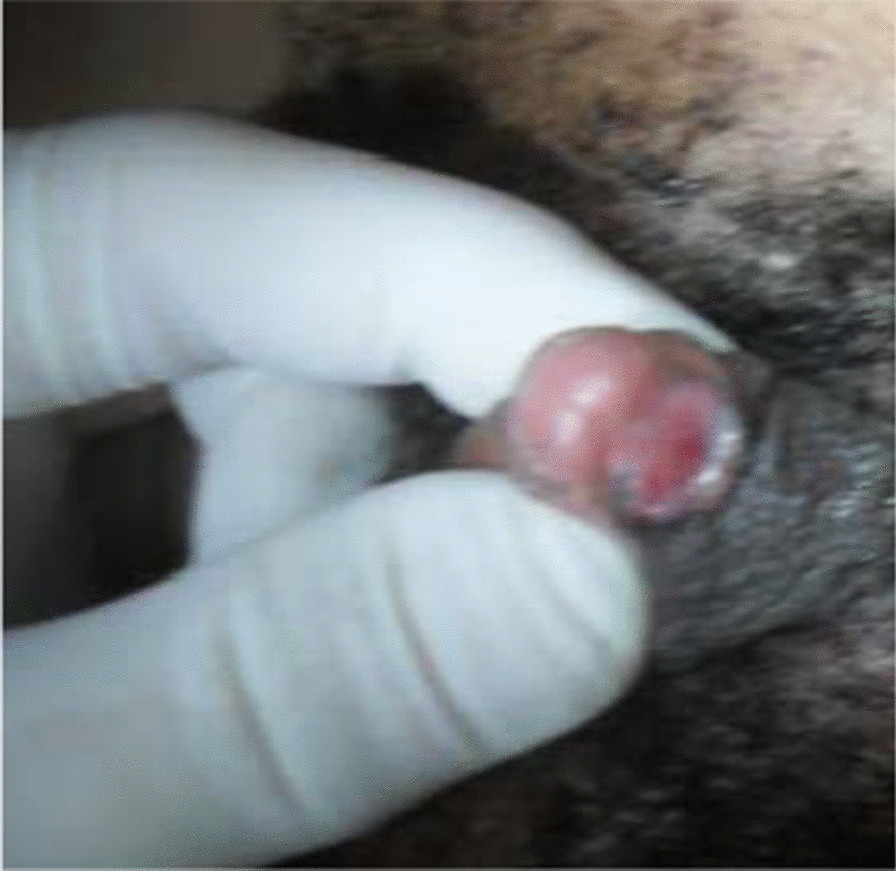
Mega clitoris with hypospadias

A 24-year-old from a majority ethnicity of Volta region Ghana was referred on account of 7 years cyclical total painless hematuria, which lasted about 2–3 days per episode for the past 3 years. This was often preceded by mild lower abdominal pain and malaise. There was no identifiable risk factor for bladder carcinoma (habits or exposure). The patient is the eldest of five siblings, who are all alive and had normal pregnancy and delivery periods. The mother was on the routine antenatal medications as always prescribed in Ghana. Two of her siblings also had a similar genital appearance and were undergoing medical examinations. She was christened and raised as a male from infancy to adolescence when she noticed the development of female secondary sexual characteristics (breast, changes in voice, hair distribution pattern, and general hip enlargement). She stopped her education and migrated to the city where she has been residing since then. Physical examination reveals feminine figure appearance, well-developed breast, female hair distribution pattern, and a feminine voice. She had a mega clitoris without vaginal opening (Fig. 1), and the vagina or labial/scrotal area shows a solid perineal mass. The urethral opening was underneath the clitoris (Fig. 2).

### Management of the case

**Fig. 3:**
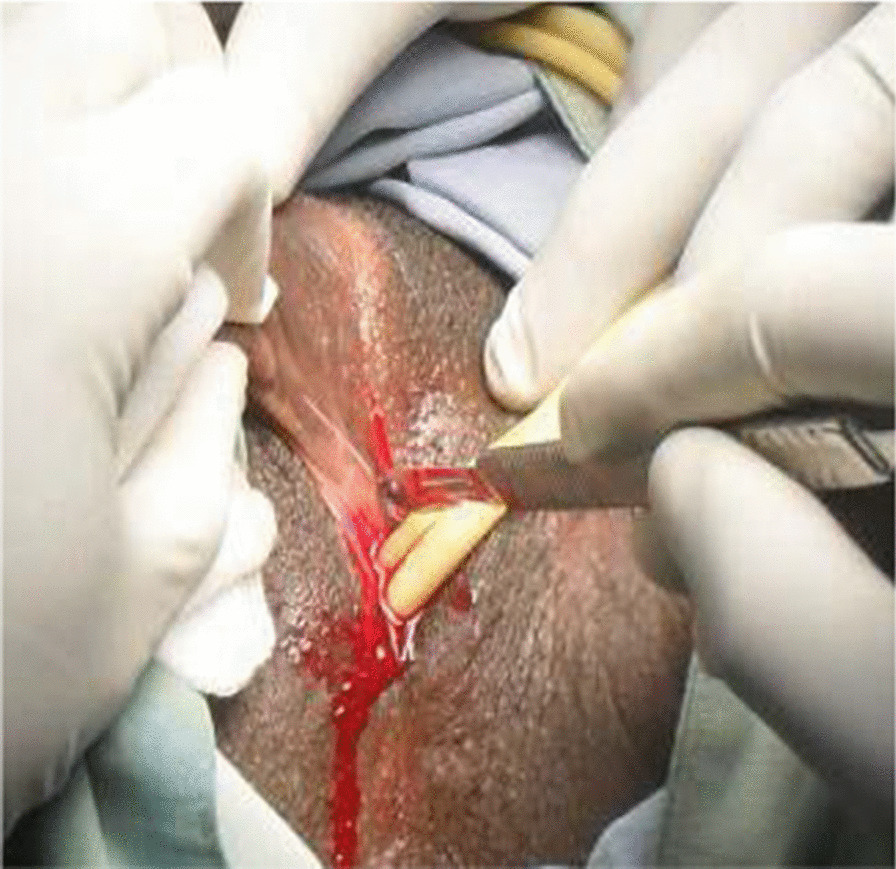
Vaginal and urethral catheters during separation

**Fig. 4:**
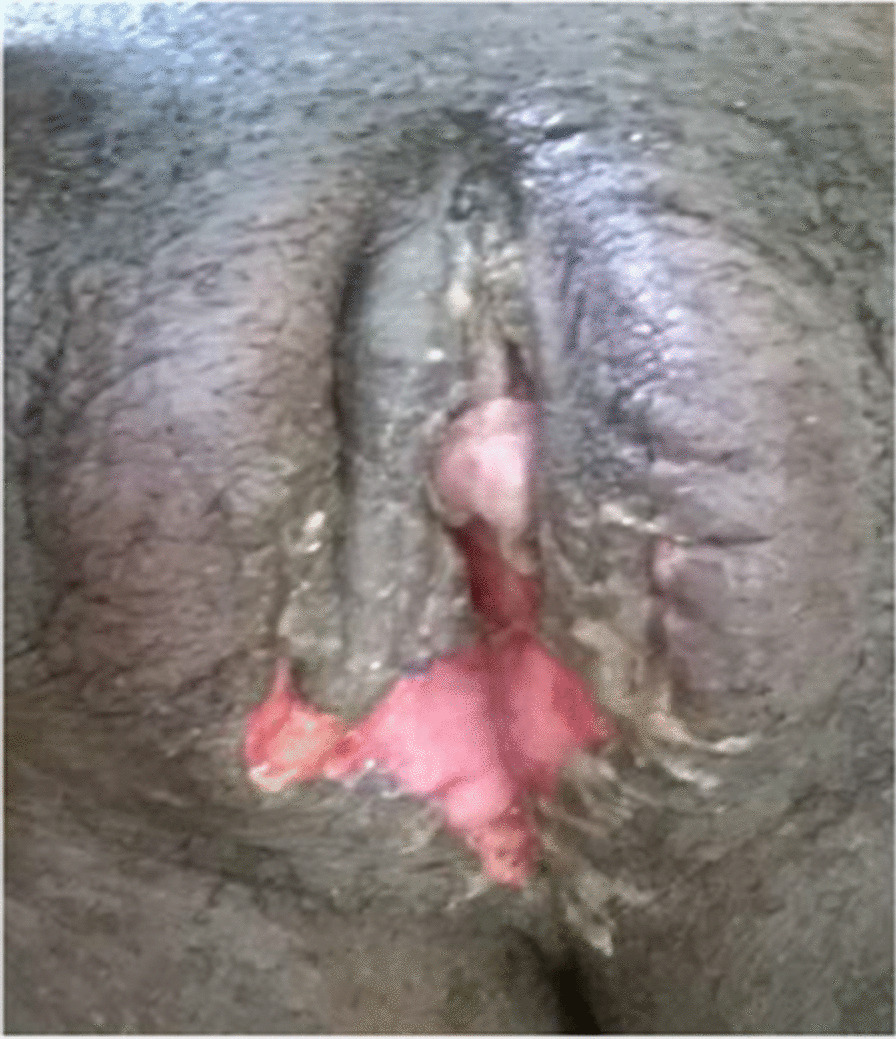
Post vaginoplasty and neoclitoroplasty

The management of the DSD in this case was focused on first ascertaining the etiological diagnosis. This was done by performing an abdominopelvic ultrasound scan, which showed normal uterus and ovaries. Urethrocystoscopy demonstrated a normal bladder mucosa, ureteric orifices, and a poorly visualized dimple in the urethral mucosa distal to the sphincter. A diagnosis of 46, XX DSD secondary to androgen excess was made (Table 1). Since all the internal female genital organs were found to be normal, the second decision of the surgical team was the assignment of the external genitalia to correspond with the internal organs; thus, construction of the vulva was indicated (Fig. 3). The client and her family were informed of the diagnosis and the treatment option arrived at by the team. Consent was given by the client and her family members in a signed form. The surgery was scheduled and duly performed with successful separation, vaginoplasty, and neoclitoroplasty, as seen in Figs. 3 and 4. The patient’s follow-ups and review after treatment were scheduled for monitoring of the aftermath of the surgery. Her review revealed that the procedure had been successful, with no adverse event. Understanding and consent were sought from the patient for the purposes of using her images for teaching, scientific publication, and demonstrations.

## Discussion

The formation of a typical male or female sex organ involves numerous and complex genetic and physiological events such as sex determination during fertilization, development at the gonadal ridge, expression of the mesonephric or paramesonephric ducts, differentiation into internal and external organs during the zygotic phase, growth of these reproductive organs to phenotypic recognition, and masculinization or feminization from birth to puberty under the influence of hormones and transcription factors [10, 11].

Managing an adult with ambiguous external genitalia requiring surgical intervention is extremely rare as patients with various spectra of the disease requiring surgical intervention most probably would have received it during childhood or adolescence [12, 13]. The current practice for managing these diseases is an early multidisciplinary approach that often includes early surgical evaluation and possible reconstruction, at most deferring until adolescence [14]. The early approach in assigning gender is influenced by the desire to limit identity crisis, which is believed to help reduce parental anxiety, satisfy some unique sex-related cultural practices, and above all, help in the sociocultural growth of the child [14].

Advocates for deferral of surgical intervention raise concern that the influence of high maternal estrogen levels that causes clitoromegaly could be mistaken for male phallus, leading to wrong gender assignment. Continuous psychological counseling while delaying vaginoplasty is currently considered. This approach is believed to allow for tissue growth that will make surgery relatively easy. Full patient participation in the evaluation and treatment, as in this case under review, is critical. The optimal timing of the surgery remains unclear. This case report has taught us many lessons associated with the timing of DSD management, including:

1. It confirms the difficulty associated with early biological sex determination among ambiguous genitalia neonates.
2. It lays out the social consequences of raising a child with a wrong gender, including predisposition to bullying and mockery by peers, withdrawal from school, need for relocation, and change of name and dressing style when the secondary sex organs become prominent.
3. It emphasizes the heightened anxiety of the patient and relatives over the delay in correction and the need for prolonging psychological counseling if a delay is contemplated.
4. There is an advantage to conducting a limited investigation (concentrating on the urogenital area and internal organs) since secondary sexual features have already developed and directed the biological gender, especially in hospitals with limited diagnostic abilities.
5. It demonstrates the relative ease of surgical intervention as a result of well-developed structures at the perineal area for dissection and raising of flaps during vaginoplasty and neoclitoroplasty.
6. It shows reduced postoperative complications in terms of infections and reconstructive breakdown.
7. It resulted in good functional outcome and patient satisfaction.

## Conclusion

The advantages of deferring surgical reconstruction with psychological counseling after early assessment need to be considered to prevent inappropriate gender assignment.

## Data Availability

The published information is available from the corresponding author on reasonable request.

## Abbreviations

DSDs: Disorders of sex development
CAH: Congenital adrenal hyperplasia
